# Assessment of personality functioning in psychosomatic medicine

**DOI:** 10.1007/s00508-021-01993-x

**Published:** 2022-03-28

**Authors:** Jolana Wagner-Skacel, Franziska Matzer, Alexandra Kohlhammer-Dohr, Nina Dalkner, Emanuel Jauk

**Affiliations:** 1 Department of Medical Psychology and Psychotherapy, Medical University of Graz (MUG), Auenbruggerplatz 3, 8036 Graz, Austria; 2 Clinical Psychology and Behavioral Neuroscience, Technische Universität Dresden, Dresden, Germany; 3 Department of Psychology, University of Graz, Graz, Austria; 4 Department of Psychiatry and Psychotherapy, Medical University of Graz (MUG), Graz, Austria

**Keywords:** Structural integration, Assessment, Somatic disease, Coping strategies, Self-regulation

## Abstract

**Background:**

Personality functioning, also referred to as structural integration, describes basic emotion-related perception and regulation capacities directed towards the self and others. Patients with impairments of personality functioning experience difficulties in self-regulation and interpersonal relations. Although personality functioning has become increasingly important in psychotherapeutic and psychiatric diagnoses and treatment planning, there is little systematic evidence on the role of personality functioning in patients with chronic and somatic diseases. This article reviews empirical studies using standardized assessments of personality functioning in patients with chronic and somatic diseases and discusses the role of personality structure in psychosomatic medicine.

**Results:**

Currently, there are only a limited number of studies using standardized assessments of personality functioning in patients with chronic or somatic diseases. The available evidence points to correlations of personality functioning with pain perception and the development of chronic pain. In addition, patients with lower levels of personality functioning may have difficulties in managing chronic conditions that require enduring changes in health behavior, such as in diabetes or posttransplantation therapy.

**Conclusion:**

The review suggests a systematic link between personality functioning and health behavior in patients with chronic diseases that relate to self-regulation and coping strategies. These findings underline the importance of assessing personality functioning for diagnostics and treatment planning in psychosomatic medicine. Finally, an assessment of personality functioning could be helpful in choosing specific psychotherapeutic treatment strategies; however, more empirical studies are needed to comprehensively prove these assumptions.

## Introduction

Patients in consultation-liaison psychiatry, psychotherapy, and psychosomatics are often regarded to be difficult to treat. Their impairments in the realms of emotion regulation and interpersonal relationships, frequently combined with an unhealthy lifestyle and few adaptive health-related behaviors, lead to challenges for healthcare professionals.

## The concept of personality functioning and structural integration

Personality functioning—amongst others also referred to as structural integration or personality organization [1]—describes basic self-related and other-related affect-laden processing and regulation capacities. Patients suffering from impairments in personality functioning—at worst resulting in personality disorder [2]—show significantly reduced psychosocial functioning, which includes difficulties in self-regulation and the regulation of interpersonal relationships. Personality functioning is assumed to vary on a continuum ranging from non-impaired/well-integrated to severely impaired/disintegrated levels [2–4]. The severity of personality pathology is defined by the degree of disturbances in self and interpersonal functioning [5]. Personality functioning on a well-integrated level is characterized by a coherent sense of self, flexible functioning even when stressed by external or internal conflicts, appropriate expression and regulation of impulses and emotions, internalized moral values and engagement in satisfying relationships [4, 5]. Individuals at lower levels of personality functioning typically display, amongst others, characteristic problems in self-regulation or self-other differentiation (i.e., the attribution of mental states to the self or another person), which are accompanied by a range of challenges related to adverse health behaviors and interpersonal relations, including the doctor-patient relationship [6]. In the clinical setting, patients with lower levels of personality functioning are often experienced as “difficult to treat” [7]. Consequently, the focus on domains beyond symptoms, such as personality functioning, has been emphasized as being highly important for indications and treatment planning [8]. Despite the theoretical and practical significance of the personality functioning concept, it is not yet commonly integrated into diagnostic and treatment processes.

The concept of personality functioning is represented in a range of contemporary models for the description of variation in personality and personality pathology, including the DSM‑5 alternative model of personality disorders (AMPD, [2]) and the upcoming ICD-11 dimensional personality disorders model [9], the operationalized psychodynamic diagnosis (OPD) levels of structural integration axis (LSIA, [4]), or the model of personality organization proposed by Kernberg et al. with the corresponding structured interview of personality organization (STIPO, [3]). Beyond that, similar concepts are represented in nonclinical personality models aiming to describe variation in basic emotion-related processing and regulation capacities (here referred to as emotional intelligence, or competence), pointing to the ubiquity of the concept across different research and applied traditions [10]. All of these models share the idea that personality functioning, structural integration, personality organization, or emotional competence, describe fundamental emotion-related capacities underlying more specific aspects of personality. These fundamental aspects are discernible from (although not independent of) more specific patterns of experience and behavior describing the individual’s propensity to feel and act in a particular manner [10]. This latter aspect is, depending on the psychological tradition, either described in terms of prevailing *conflicts* (such as in the OPD system) or *personality traits* (such as in the DSM‑5 and ICD-11 systems). For instance, a narcissistic personality style would be regarded as a manifestation of a prevailing conflict in the OPD system (conflict within the self-esteem system [4]) and combination of descriptive traits (facets of antagonism) in the DSM‑5 system [2]. The level of intrapersonal and interpersonal functioning on which the—in this example narcissistic—individual operates would be considered an indicator of personality functioning, ranging from no impairment to severe impairment [2, 8]. In this line of thinking, it is thus the level of personality functioning (the A criterion in the DSM‑5 AMPD, [2]), rather than the more specific aspects of personality, that informs about the severeness of impairments.

Personality functioning is commonly assessed by trained clinicians or researchers using standardized or semi-standardized interviews, such as the previously mentioned STIPO [3], the OPD interview [4, 8] or the structured clinical interview for the AMPD [11]. All of these have been found to be reliable and valid diagnostic assessments, which predict an array of general clinically relevant outcomes, such as number of diagnoses, symptom load and global functioning [8]. Expert interviews can be complemented by self-report measures of personality functioning, which have been developed for all systems (e.g. [12]).

Historically, the dimension of personality functioning has been part of psychoanalytic and psychodynamic theory and research since Sigmund Freud presented his first structural model in 1900 [13]. Later on he defined health as being able to love and to work. These capacities can be regarded as the precursors of what we now call personality functioning; the functions of the ego help an individual adjust and adapt to his or her reality. Personality functioning becomes apparent in the shape of capacities of the self. These include self and other recognition, regulation, communication, and attachment. The interaction within the parent-child dyad as our first environment—as an intersubjective encounter that predisposes the development of self and the other—modulates the organization of our body-mind interoceptive and exteroceptive connections in relation to the other [14]. Our environment is bound to what we experience; we change in the light of the picture we make of ourselves. Otto Kernberg developed an influential systematic model of personality functioning, for which he coined the term personality organization [15]. According to his model, personality organization is reflected through five domains of functioning: the *coherence of identity*, the *quality of object relations*, the *maturity of defense mechanisms, aggression*, and *moral values* [15]. Based on these domains of functioning, Kernberg differentiates between a healthy/mature, a neurotic, a borderline, and a psychotic personality organization [16]. A similar model is implemented in the OPD system, where the LSIA incorporates theories from ego-psychology, self-psychology, object relations theory and developmental psychology in terms of core capacities in *perception, regulation, communication* and *attachment* [17]. Each of these core capabilities can be directed towards the self and towards others, leading to a total of eight dimensions (see Table 1). A similar model has been established in the DSM‑5 AMPD, where the two large domains *self* and *interpersonal functioning* are parted in (roughly speaking) *perceptual* (identity and empathy) and *regulatory* (self-direction and intimacy) aspects (see Table 1). Finally, a model has been proposed for the upcoming ICD-11 ([9], see also [18]). To sum up, several contemporary models of personality functioning assume that self-related and other-related affect-laden information processing and regulation capacities make up the basis on which more nuanced personality characteristics operate. This basis ranges from well-integrated with no impairment to disintegrated structures with severe impairment in functioning, which is of high diagnostic relevance for mental and also physical health, as discussed in the following.

**Table 1:** Comparison between subscales of the level of structural integration axis (LSIA) of operationalized psychodynamic diagnosis (OPD) and DSM‑5 level of personality functioning scale (LPFS)

	DSM‑5		OPD‑2	
Self	Identity	– stable and coherent self – stable self-esteem – capacity to experience, regulate, tolerate affect	Self-perception	Self-perception
				Affect differentiation
				Sense of identity
			Self-regulation	Affect tolerance
				Impulse control
				Regulation of self-esteem
	Self-direction	– goal pursuit – utilization of internal standards of behavior – self-reflection	Emotional communication: internal	Experiencing affect
				Use of fantasies
				Bodily self
			Attachment to internal objects	Internalization
				Use of introjects
				Variability of attachment
Other	Empathy	– understanding others’ experiences and motivations – tolerance of differing perspectives – understanding of social causality	Object perception	Self-object differentiation
				Holistic object perception
				Realistic object perception
			Regulation of relationships	Protecting relationships
				Balancing interests
				Anticipation
	Intimacy	– connection with others – desire and capacity for closeness – cooperative behavior	Emotional communication: external	Establishing contact
				Communicating affect
				Empathy
			Attachment to external objects	Capability for attachment
				Accepting help
				Detaching from relationships

## Personality functioning in psychiatry

The assessment of personality functioning has become increasingly important in psychiatric diagnostics for indication and treatment planning [19]. As mentioned before, the impairment of personality functioning represents the primary criterion of the DSM‑5 AMPD. A recent study investigated emotional experiences in patients with major depressive disorder, comparing groups with and without comorbid borderline personality disorder to each other. It was investigated whether depression severity or personality functioning would mediate group differences, and which aspects of emotional experience change during psychotherapy. Lower levels of personality functioning in depressed patients with borderline personality disorder (BPD) were associated with a broader spectrum of negative emotions [20].

In patients with bipolar disorder, a low level of personality functioning and an insecure attachment style was accompanied by a significantly higher symptom load [21].

Schneider et al. compared male and female patients with respect to the OPD‑2 system, treatment variables and outcome after multimodal psychodynamic inpatient psychotherapy. There were differences between men and women in the main diagnoses, with a higher proportion of eating disorders and a longer treatment duration among women. Therapy discontinuation rates were low and did not differ between the sexes. Overall, both sexes seem to benefit equally from psychodynamic inpatient psychotherapy in terms of symptom improvement [22].

## Personality functioning in psychosomatic medicine

According to biopsychosocial medicine, psychosocial aspects may be relevant in pathogenesis, triggering or maintaining multiple somatic symptoms and diseases. Personality functioning may therefore be one relevant factor contributing to health and disease. Reduced personality functioning goes along with reduced core capacities in perception, regulation, communication, and attachment; for example, there are deficits in experience, verbal expression, and regulation of emotion [23]. Such impairments in self and interpersonal functioning may impact on coping strategies, emotion regulation and stress [24, 25]. Via stimulation of stress-regulating systems like the hypothalamic-pituitary-adrenal axis or the autonomic nervous system, but also via alterations of the immune system, a chronic activation of these systems results in their dysregulation and may finally lead to aggregate physiological and mental consequences [26, 27]. We therefore suggest that there is a link between personality functioning and physical as well as mental health, including affective dynamics such as depressive, anxiety, and somatization symptoms in patients with somatic diseases [28]. Interrelations between personality functioning and health might also be mediated via adverse health behavior and lifestyle [29]. Furthermore, problems in forming and regulating interpersonal relationships including the doctor-patient relationship may impact on the course of diseases and adherence to treatment [7].

In the following sections, studies assessing personality functioning in various medical fields are reviewed. Starting from the field of internal medicine, we will continue with the phenomenon of chronic pain and development of postoperative pain and finally review studies from the field of transplantation medicine.

## Personality functioning in medical fields

### Endocrinology

Psychosocial variables influence chronic diseases. Ehrenthal et al. investigated the associations of depression and personality functioning with glucose regulation in patients with type 2 diabetes [7]. All patients with first diagnosed type 2 diabetes were monitored over a period of 36 months in plasma glucose concentration (HbA1c), body mass index, and personality functioning using the operationalized personalized psychodynamic OPD structure questionnaire (OPD-SQ [12]), a self-report measure of personality functioning. In a sample of 70 patients, a standardized disease-management program resulted in a significant decrease of HbA1c over a period of 6 months. Participants with higher impairments in personality functioning showed less decline in HbA1c scores during 6 months. Depressive symptoms were not associated with levels of HbA1c [7].

As in other chronic diseases, the risk factors, symptoms and subsequent long-term damage in diabetes mellitus can be managed through lifestyle changes or adherence to the treatment [30]. Adherence depends on the patients’ emotional and cognitive abilities influenced by personality and mental states or traits. Individuals with type 2 diabetes have higher levels of depressive symptoms [31]. Distress and coping mechanisms influence the association of type 2 diabetes with depression, and depressive symptoms in the other direction increase the risk of developing type 2 diabetes [32]. Impairments in personality functioning may be harmful via changes in health-behavior and a resulting lack of adherence. Even mild impairments in a person’s personality functioning may interfere with the capacity to implement and maintain behavioral changes.

### Eating disorders and obesity

In a recent study, personality functioning as assessed by the OPD-SQ was found to discriminate between subtypes of eating disorders [33]. Patients with anorexia nervosa purging type demonstrated the highest impairments in personality functioning, whereas patients with anorexia nervosa restricting type showed a higher level of personality functioning compared to patients with bulimia nervosa and anorexia nervosa purging type. The largest differences included self-perception, object perception, and attachment to internal objects. These exploratory findings suggest that the OPD-SQ could be useful in clinical assessment and classification of patients with eating disorders. Besides, different psychotherapeutic interventions should be used to treat patients with different types of eating disorders.

Based on the biopsychosocial and economical association of obesity and personality traits, there is a critical discussion of understanding the development of overweight and obesity [34]. Personality traits can be important risk or protective factors in the development of obesity. A recent study among obese women with binge eating disorder found impairments in both personality traits and personality functioning as compared to obese and non-obese community controls [35]. Obese patients with binge eating disorder had a significantly more vulnerable personality profile with impairments at almost all levels of maladaptive personality functioning. In addition, obese patient groups (with and without binge eating disorder) had lower levels of adaptive personality functioning. The additional assessment of personality functioning could therefore provide a new perspective in understanding the relationship between obesity, binge eating disorder, and personality and give implications for the successful treatment of patients concerned.

### Chronic pain

A study of Fischer-Kern et al. investigated the relationship between psychiatric classification and personality functioning of chronic pain patients, as assessed by the STIPO. The most severe impairments in personality functioning were found in the STIPO dimensions rigidity, identity, primitive defenses and coping [36]. The investigation of structural aspects of personality in chronic pain patients could be central for diagnostics and treatment planning with an impact on emotional and social functions. A recent meta-analytic review by Burke et al. [37] demonstrated that individuals with chronic pain reported experiencing severe problems in numerous psychological domains including anger/hostility, self-efficacy, self-esteem and general emotional functioning; however, more studies are definitively needed to investigate the role of personality functioning in patients with chronic pain [37].

### Orthopedics

In orthopedics, the role of personality functioning has recently been studied among patients after total knee arthroplasty [38]. Total knee arthroplasty is a common treatment for end-stage knee osteoarthritis and although pain relief can be achieved for most patients, psychological factors such as poor mental health and pain catastrophizing have been shown to negatively affect postoperative outcome [39]. In addition, borderline personality features have been linked to a higher prevalence of osteoarthritis [40]. In a pilot study among 47 patients with osteoarthritis undergoing total knee arthroplasty [38], personality functioning was assessed using the inventory of personality organization (IPO, [41]), a self-report version of the previously mentioned structured interview of personality organization (STIPO [3, 6]). Pain levels before and 8 weeks after total knee arthroplasty as well as pain change were assessed and applied as dependent variables in a subsequent backward linear regression analysis including several predictors, such as an observer-rated knee questionnaire, the brief symptom Inventory including the subscales depression, anxiety, somatization, a global severity index of mental health as well as personality functioning including the IPO subscales *identity diffusion, primitive defenses, reality testing*, and a total score. Results showed that self-rated postoperative pain, that could be rather classified as acute pain, was significantly predicted by the IPO subscale identity diffusion; interestingly, higher identity diffusion predicting less pain. The authors concluded that this finding matches a theory by Sansone and Sansone [42] known as the pain paradox in borderline personality disorder, suggesting that patients with BPD tend to have lower sensitivity for acute painful stimuli and higher sensitivity to chronic pain. The surgery itself might represent a physical trauma within an interpersonal relationship, thus evoking a psychodynamic reaction affecting disintegrated bodily aspects of the self and by that way altering pain sensitivity. Thus, screening for personality functioning in patients undergoing total knee arthroplasty and specific psychotherapeutic support might be useful for at least some patients.

In a next study by the same study group [43], 144 patients scheduled for total knee arthroplasty were enrolled and tested for personality structure using the IPO. Pain and function of the knee were assessed before and 12 months after the surgery. A stepwise multiple linear regression included the predictors age, sex, the subscales and total score of the IPO, the baseline self-rated knee pain and function, an observer-rated knee index and a categorical variable for the presence or absence of BPD. Analyses estimated the significant predictors of knee pain 12 months after surgery. Postoperative pain 12 months after surgery, classified as chronic pain, was predicted by preoperative knee function, female sex and the subscale primitive defenses of the IPO indicating more primitive defenses predicting more pain. In accordance with previous findings, aspects of borderline personality organization, such as primitive defenses could account for vulnerability for the development of chronic pain. These results suggest certain psychodynamic and psychosomatic mechanisms of maladaptation after total knee arthroplasty in some patients, who might not be able to cope with the affects, inner conflict, tension and eventually reactivation of experienced trauma evoked by such a surgical intervention. Consequently, fundamental psychic defenses could manifest as bodily pain.

### Transplantation medicine

A study of Calia et al. evaluated the association between attachment style, compliance, quality of life, and renal function in adult patients after kidney transplantation [44]. Although this study did not directly assess personality functioning, it included measures of alexithymia, emotional self-efficacy and attachment, which are all considered part of personality functioning/structural integration. Patients with avoidant attachment had a significantly better perception of their own general health than patients with anxious attachment. Calia et al. suggested that the evaluation of the attachment style in adult kidney transplant patients can increase compliance with goal-directed psychological support program for these patients [44]. In another study, they evaluated the associations between alexithymia or emotional self-efficacy and compliance in renal transplant patients. Patients with high levels of alexithymia reported a negative perception of their quality of life (QoL) and lower levels of compliance compared with patients with low levels of alexithymia. The ability to recognize and express emotions as well as the strongly related management of negative emotions may influence compliance and QoL of renal transplant patients. Therefore, psychological support could be useful in these patients in order to increase their compliance and QoL [45].

## Borderline personality disorder in psychosomatic medicine

As the available systematic evidence on the role of personality functioning is psychosomatic medicine is limited, we complement our review by a short discussion on findings on BPD in this field. The BPD, also known as emotionally unstable personality disorder, is a severe mental disorder with significant impairments in personality functioning. Common problems include difficulties in regulating emotions and impulses, intense and unstable interpersonal relationships and an inconsistent sense of self. In the DSM‑5 model of personality disorders, general criteria and the severity of personality disorders are closely linked to such impairments in self and interpersonal functioning. From a psychodynamic point of view, it is assumed that pathology and severity of personality disorders in general and of BPD in particular are determined by the degree of structural impairment. Therefore, BPD reflects the single personality disorder diagnosis which most closely resembles impairments in general personality functioning, rather than specific traits (such as for instance narcissistic or histrionic personality disorders). Although a BPD diagnosis and a personality structure at the borderline functioning level are not identical, they are largely overlapping. For example, a study among a sample of 104 patients with a clinical diagnosis of BPD and an assessment of personality organization using the STIPO revealed that all patients were classified at a level of borderline personality functioning. Furthermore, more clinically severe forms of BPD were linked to a lower level of personality functioning [46].

### Somatic comorbidity in BPD

In psychosomatic medicine, the higher prevalence of several somatic conditions among patients with BPD is of particular importance [47]. This can already be seen among remitted BPD patients: Zaharini et al. [48] compared 64 patients with BPD with a group of 200 participants with a former diagnosis of BPD. Results showed that several medical conditions, such as syndrome-like conditions (chronic fatigue, fibromyalgia, temporomandibular joint syndrome), obesity, osteoarthritis, diabetes, hypertension, chronic back pain and urinary incontinence were significantly more common among non-remitted borderline patients as compared to remitted borderline patients. In general, several somatic illnesses have a higher prevalence when compared to community samples [49, 50]. These include cardiovascular diseases, metabolic conditions and gastrointestinal diseases, venereal diseases, Human Immundeficiency Virus (HIV), urinary incontinence and sleep disorders. Moreover, skin conditions, rheumatoid arthritis and the outcome after plastic surgery have been addressed. As already mentioned, disturbances in the regulation of pain sensation and pain state also regularly occur among this group. A recent review by Doering gives an overview over BPD in patients with medical illnesses [47].

### Health-related lifestyle

The BPD patients report more unhealthy lifestyle choices [51], including smoking, daily alcohol use, lack of regular exercise, daily use of sleep medication or overuse of pain medication. A misuse or abuse of prescriptions and medications, such as analgesics and high-potency benzodiazepines is also common in BPD [51].

### Healthcare utilization

Finally, costly forms of treatment, such as medically related emergency room visits or medical hospitalizations are also more frequent among patients with BPD [48]. In a review by Sansone and Sansone [52] on BPD in the primary care setting, the authors reported higher rates of healthcare utilization among this group. This might be related to the tendency of some BPD patients to develop high somatic preoccupation and/or symptoms of somatization. The same authors also showed that patients with BPD in the primary care setting tended to present with unsubstantiated chronic pain as well as somatic preoccupation as compared to BPD patients in mental health settings [49].

### Illness perceptions

Misperceptions of one’s illness have also been related to BPD. For example, BPD patients with diabetes were found to have negative distortions or a sense of disability in illness perceptions, thus perpetuating the victim role [50]. In general, BPD goes along with a negative perception of health which, in consequence, might impact on health-related behaviors, compliance, and healthcare utilization.

## Outlook: neuroscientific aspects of personality structure

It has been proposed that one way of bringing psychodynamic concepts and neuroscience together could be via the OPD, as this instrument provides both an expanded view of individual psychological content and a systematic reduction of content that is necessary for experimental settings [53]. That way, it would also serve as an option to individualize experiments [54]. The authors propose that “only if the experiment touches the mentally represented themes that are of individual relevance to each subject, results could have validity and meaning in a deeper sense” [55]. The OPD could therefore be used for gathering individualized information in a systematic way and it could also be helpful in interpreting brain activity on a psychodynamic level. For example, in such an individualized paradigm Kessler et al. presented individual stimulus sentences that had been gathered via OPD to 29 healthy female subjects who freely associated to these stimuli while being in an Magnetic Resonance Imaging (MRI) scanner [56]. Associations to conflict-related sentences were associated with several behavioral and psychophysiological correlates that correspond with the concept of repression, a central defense mechanism in psychodynamic theory. Recently, a neurobiologically and clinically grounded model of personality organization has been introduced. This so-called neuropsychodynamic model relates the psychodynamic model of personality functioning, the construct of the self and its neuronal correlates to each other [57]. Empirical data on neuronal correlates of the self suggest that early relational and attachment experiences as well as the brain’s resting state activity relate to the concept of the self. The authors propose a multilayered model of the self, with four different layers (relational alignment, self-constitution, self-manifestation, and self-expansion) that are associated with different neuronal correlates, corresponding to different levels of personality organization including neurotic and borderline organization. That way, the psychodynamic concept of personality organization could also be linked to the concept of the self and its neuroscientific correlates; however, future research will show if this novel neuropsychodynamic model of personality organization will find further empirical support.

## Conclusion

Only a limited number of studies in psychosomatic medicine included an assessment of personality functioning so far. Whereas for patients with borderline personality disorder (BPD) several studies confirmed high somatic comorbidity, unhealthy lifestyle choices, negative perception of health and adherence problems, little is known about patients whose impairments in personality functioning do not meet the criteria of a diagnosis of personality disorder. Regarding pain perception, chronic pain was found to be associated with a lower level of personality functioning [36]. While acute postoperative pain was lower in patients with impairments in personality functioning, chronic postoperative pain was predicted by lower personality functioning [43]. Health conditions such as diabetes or after organ transplantation, that require enduring changes in health behavior, might be difficult to manage for patients with impairments in personality functioning [7]. While this might be experienced as a lack of adherence in the clinical encounter, an important implication of the reviewed studies for clinicians is to consider impairments in personality functioning as a potential source of adherence problems in their patients. In eating disorders, an assessment of personality functioning could be used for selecting appropriate psychotherapeutic treatment strategies, as some subtypes of eating disorders are associated with different levels of structural integration [33].

In the psychiatric as well as in the somatic setting, patients with impairments of personality functioning present with self-regulation disturbances and relationship difficulties. The assessment and characterization of personality and attachment styles may be of particular value in identifying individuals who may respond to certain forms of psychotherapeutic treatment. These patients may need more information about their illness and medication; they may benefit from more frequent appointments and a more proactive attitude of the therapist or doctor. The self-awareness of patients with impairments in personality functioning should be improved by therapeutic interventions targeting self-awareness and teaching strategies and skills for regulating emotions and relationships. Finally, they may benefit from interventions designed to foster a healthy lifestyle.

Summing up, results so far underline the importance of assessing personality functioning for diagnosis and planning of psychotherapeutic treatment for somatically ill patients. An assessment of personality functioning could be helpful in several fields of psychosomatic medicine including therapy of chronic pain and adherence to treatment in chronic conditions; however, more empirical studies are needed to prove the appropriateness of these assumptions.
